# Algal-Derived Halogenated Sesquiterpenes from *Laurencia dendroidea* as Lead Compounds in Schistosomiasis Environmental Control

**DOI:** 10.3390/md20020111

**Published:** 2022-01-29

**Authors:** Guilherme Senna dos Santos, Patrícia Aoki Miyasato, Erika Mattos Stein, Pio Colepicolo, Anthony D. Wright, Carlos Alberto de Bragança Pereira, Miriam Falkenberg, Eliana Nakano

**Affiliations:** 1 Laboratory of Parasitology, Butantan Institute, São Paulo 05503-000, SP CEP, Brazil; gsennasantos@gmail.com (G.S.d.S.); patricia.aoki@butantan.gov.br (P.A.M.); 2 Biochemistry Department, Chemistry Institute, USP—Universidade de São Paulo, São Paulo 05508-00, SP, Brazil; stein.erika.m@gmail.com (E.M.S.); piocolep@iq.usp.br (P.C.); 3 DKI College of Pharmacy, University of Hawaii at Hilo, Hilo, HI 96720, USA; adwhawaii@gmail.com; 4 Right Consulting, 15 Amauulu Road, Hilo, HI 96720, USA; 5 Institute of Mathematics and Statistics, USP—Universidade de São Paulo, São Paulo 05503-000, SP CEP, Brazil; cadebp@gmail.com; 6 Department of Pharmaceutical Sciences, Federal University of Santa Catarina, Florianópolis 88040-970, SC, Brazil; miriam.falkenberg@gmail.com

**Keywords:** marine natural products, terpenes, *Schistosoma mansoni*, *Biomphalaria*, larvicide, molluscicide, (−)-elatol, rogiolol, obtusol

## Abstract

Schistosomiasis has been controlled for more than 40 years with a single drug, praziquantel, and only one molluscicide, niclosamide, raising concern of the possibility of the emergence of resistant strains. However, the molecular targets for both agents are thus far unknown. Consequently, the search for lead compounds from natural sources has been encouraged due to their diverse structure and function. Our search for natural compounds with potential use in schistosomiasis control led to the identification of an algal species, *Laurencia dendroidea*, whose extracts demonstrated significant activity toward both *Schistosoma mansoni* parasites and their intermediate host snails *Biomphalaria glabrata*. In the present study, three seaweed-derived halogenated sesquiterpenes, (−)-elatol, rogiolol, and obtusol are proposed as potential lead compounds for the development of anthelminthic drugs for the treatment of and pesticides for the environmental control of schistosomiasis. The three compounds were screened for their antischistosomal and molluscicidal activities. The screening revealed that rogiolol exhibits significant activity toward the survival of adult worms, and that all three compounds showed activity against *S. mansoni* cercariae and *B. glabrata* embryos. Biomonitored fractioning of *L. dendroidea* extracts indicated elatol as the most active compound toward cercariae larvae and snail embryos.

## 1. Introduction

Helminths are the most common infectious agents affecting human populations in developing countries. Over one billion people in sub-Saharan Africa, Asia, and the Americas are infected with one or more species of helminth species, imposing a significant burden on public health [1].

Ranking second among the helminthiasis, schistosomiasis affects almost 240 million people worldwide [2]. The transmission cycle of these infectious agents requires contamination of surface water by excreta, specific freshwater snails as intermediate hosts, and human–water contact [3]. Currently, the chemical arsenal for schistosomiasis control is limited to two compounds. Praziquantel is the only available drug for the treatment and control of schistosomiasis [4], while niclosamide is the only recommended molluscicide to control the host snail populations [5]. The WHO agenda for neglected tropical diseases sets a global milestone of 2030 for the elimination of schistosomiasis as a public health problem by increasing the mass administration of praziquantel in order to reach 75% treatment coverage [6]. Such widespread use of this one drug clearly raises the issue of drug resistance. Furthermore, since chemotherapy alone is not able to stop transmission, additional interventions must be integrated into programs aiming to reduce reinfection, lower prevalence, and move toward elimination [7]. The implementation of snail control as part of an integrated control and elimination program has been reinforced with the inclusion of schistosomiasis in the WHO resolution of reducing the incidence of vector borne diseases by at least 40% by 2025, and developing new molluscicides [8].

Recently, we detected the in vitro activity of seaweed extracts against *S. mansoni*. Three out of the thirteen tested extracts induced lethal effects in the exposed worms [9]. In a more comprehensive screening, in addition to the effects on *S. mansoni*, the molluscicidal activity was evaluated, indicating the activity of 22 out of 37 species in at least one of the models [10]. In the present study, the halogenated sesquiterpenes (−)-elatol, obtusol, and rogiolol isolated from the extract of one of the 22 species, *Laurencia dendroidea*, were assayed for their antischistosomal and molluscicidal activities. Additionally, *Laurencia dendroidea* extracts were submitted to monitored fractioning for molluscicidal and cercaricidal activity.

## 2. Results and Discussion

### 2.1. Screening of Isolated Sesquiterpenes for Antischistosomal and Molluscicidal Activities

Found as common constituents in *Laurencia* extracts in different combinations, halogenated sesquiterpenes have been tested for different biological activities [11,12,13,14,15]. The majority of studies have focused on antimicrobial activity and the potential of these compounds as therapeutic agents. Most of the data into antiparasitic activity concern protozoans [16]. Data on anthelmintic activity are scarce [17,18,19], and so far, there are no studies on the antischistosomal activity of algal-derived compounds.

**Figure 1:**
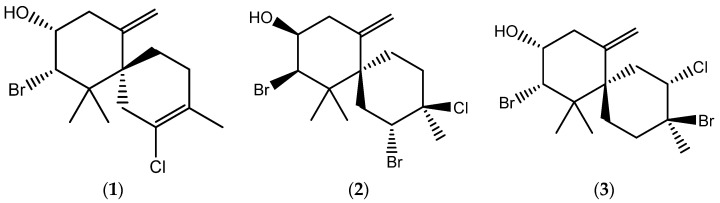
Seaweed-derived halogenated sesquiterpenes: (**1**) (−)-elatol; (**2**) (−)-rogiolol; (**3**) (+)-obtusol.

The halogenated sesquiterpenes (−)-elatol (**1**), (−)-rogiolol (**2**), and (+)-obtusol (**3**) (Figure 1), purified by repeated HPLC on silica gel guided by NMR, from *Laurencia dendroidea* were screened for antischistosomal activity on *Schistosoma mansoni* adult worms and cercariae, and for molluscicidal activity on *Biomphalaria glabrata* embryos (Table 1).

*S. mansoni* adult worms were exposed to a single concentration of each compound for 96 h. Rogiolol was the most active among the analogs causing 90% mortality of the exposed worms after 72 h, and 100% by the end of the exposure. Rogiolol also affected their reproduction, inducing 100% couple separation after 24 h and total inhibition of oviposition.

Obtusol caused 20% mortality for female worms; male survival was not affected. Motility was affected in all exposed worms at different levels, with females being more sensitive. Reproduction was significantly affected, with 100% separation of worm couples and total inhibition of oviposition.

(−)-Elatol had no effect on survival and minimal effect on motility. Reproduction was significantly affected with 100% separation of worm couples and total inhibition of oviposition.

*S. mansoni* cercariae larvae were exposed to 25 µg mL^−1^ of each compound for 2 h. The three analogues caused 100% mortality of larvae after 5 min of exposure.

*B. glabrata* embryos were exposed to 25 µg mL^−1^ of each compound for 24 h. At this level, all the analogues induced 100% mortality of embryos both at the blastulae and veliger stages.

The worm in vitro assay results showed that although all the three analogues induced effects on exposed worms at some level, significant differences were observed for each compound, suggesting a molecular target. Rogiolol exhibited the highest antischistosomal effect, causing 100% mortality, while (−)-elatol had no effect on survival; this specificity was not observed for *S. mansoni* cercariae and snails.

### 2.2. Bioguided Fractionation of Laurencia dendroidea Led to Elatol

**Figure 2:**
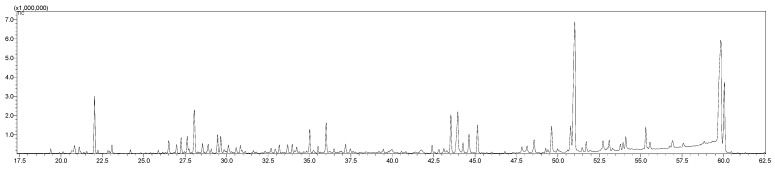
Chromatogram (18–62 min) of *Laurencia dendroidea* extract in dichloromethane obtained by GC-MS.

*L. dendroidea* extracts were previously selected for bioguided fractionation together with other algal species in our trial studies [9,10]. In the present study, extracts of *L. dendroidea*, as shown in the GC-MS chromatograms in the 20–60 min range (Figure 2), revealed samples to be complex mixtures that contained a wide variety of volatile compounds from different chemical classes according to searches of various GC-MS libraries.

**Figure 3:**
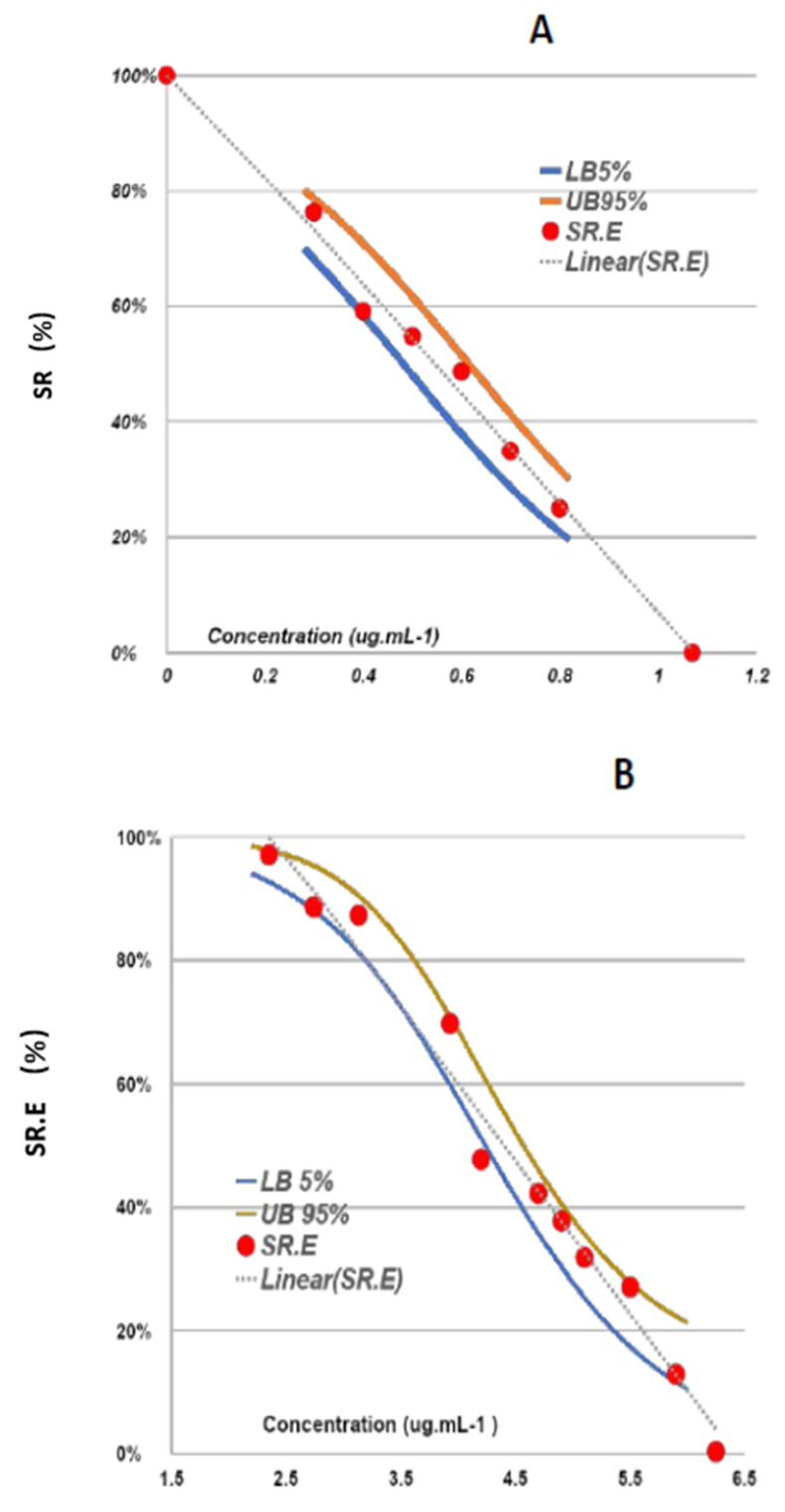
Survival of *B. glabrata* embryos exposed for 24 h to (−)-elatol at (**A**) blastulae and (**B**) veliger stages. SR = survival rates; SR.E = SR estimate; LB = lower bound, and UB = upper bound.

Bioguided fractionation of one of these extracts, employing assays for molluscicidal and cercaricidal activities, indicated (−)-elatol as the primary active metabolite of one of the active fractions and of the total extract as shown by the GC-MS peak at RT = 51 min (Figure 2). (−)-Elatol, after being isolated, was assessed for its activity toward *B. glabrata* embryos and *S. mansoni* cercariae. It was found to have significant activity toward *B. glabrata* embryos at both blastulae and veliger stages, inducing 100% mortality at 1.56 µg mL^−1^ (4.86 µM) and 6.25 µg mL^−1^ (18.73 µM). The embryos at the blastulae stage were eight times more sensitive to the compound than those at the veliger stage, with LC_50_ values of 0.55 mg L^−1^ (1.65 μM) at the blastulae stage and 4.52 mg L^−1^ (13.5 μM) at the veliger stage (Figure 3).

(−)-Elatol was tested for activity toward *S. mansoni* cercariae (Table 2) at concentrations of 3.13 to 100 µg mL^−1^. Above 12.50 µg mL^−1^ (37.46 µM), 100% mortality was observed. At 6.25 µg mL^−1^ (18.73 µM), more than 50% of the larvae showed temporary paralysis; some of them recovering motility after 15 min of exposure. After 120 min of exposure, 20% of the cercariae remained motionless at the bottom of the plates. At concentrations lower than 3.13 µg mL^−1^, no effects were observed.

According to the WHO guidelines [5], evaluation of efficacy and the determination of intrinsic molluscicidal activity of a new compound by establishing a dose–response curve is one of the main objectives of laboratory testing of molluscicidal agents. In the present study, the clear dose–response relationship for the *B. glabrata* embryos’ survival showed that (−)-elatol is a viable candidate for further testing on adult snails. The main advantage of the embryo assay at the initial stages of development is the use of as little natural material as possible.

In addition to the optimal molluscicidal activity observed in the present study, (−)-elatol showed significant cercaricidal activity. Despite the lack of standard protocols for testing cercaricidal activity in the main guidelines, some studies proposed targeting both host snails and *Schistosoma* larvae to control schistosomiasis transmission [20]. Surprisingly, unlike adult worms, the *S. mansoni* cercariae were sensitive to elatol, even at low concentrations. Effects on cercariae motility were observed from 3.13 µg mL^−1^, and at 12.5 µg mL^−1^, (−)-elatol killed 100% of cercariae.

Algal-derived products have been proposed as suitable alternatives for insecticidal agents, as they are relatively safe, biodegradable, and readily available worldwide [21]. Although cercaricidal activity of algal-derived compounds is unknown, (−)-elatol has been identified as the compound responsible for the larvicidal activity observed in *Aedes aegypti* exposed to *L. dendroidea* crude extracts [14,15].

Host snail control was the only strategy for the prevention of schistosomiasis prior to the advent of preventive chemotherapy. However, due to the low interest of public health programs in implementing vector control interventions after the development of safe drugs, snail control has declined [22].

Recently, schistosomiasis control was included in the WHO resolution to reduce at least 40% of the incidence of vector-borne diseases by 2025 [23], and schistosomiasis is targeted for elimination as a public health problem by 2030 [6]. As part of the WHO strategic approach to complement mass treatment campaigns, snail control measures are being reinforced, and the search for new cost-effective and nontoxic molluscicides was encouraged [8].

So far, there are no data on molluscicidal or cercaricidal activity of (−)-elatol or halogenated sesquiterpenes. Therefore, based on the results obtained in the present study, (−)-elatol and its derivatives are proposed as potential lead compounds to aid in the development of a new product to assist with the environmental control of schistosomiasis.

## 3. Materials and Methods

### 3.1. General Procedures

We performed 1D and 2D nuclear magnetic resonance (^1^H and ^13^C NMR, HSQC, and HMBC) spectroscopy employing a Brucker Avance III 500 MHz spectrometer equipped with a 5 mm TXI field gradient probe-head having dedicated channels for ^1^H, ^13^C, and ^15^N. Samples were prepared in deuterated chloroform (CDCl_3_) and results were analyzed with Bruker TopSpin 3.5 software. Some measurements were made with a Bruker Avance DRX 400 MHz NMR spectrometer equipped with various probe-heads and TopSpin version 2.1 software (Bruker BioSpin, Billerica, MA, USA).

Optical rotation data were collected using a Rudolph Research Analytical Autopol IV Automatic polarimeter (Hackettstown, NJ, USA) or Jasco DIP-370 Digital Polarimeter (Hachioji, Tokio, Japan). IR spectra were measured using a Thermo Scientific Nicolet iS10 FTIR spectrophotometer fitted with a Smart iTR (Waltham, MA, USA) or an Agilent Cary 630 FTIR controlled by MicroLab PC software (Santa Clara, CA, USA).

### 3.2. Algae Sampling

*Laurencia dendroidea* J. Agardh samples were collected at Ubu and Castelhanos beach, Anchieta (20°48′6″ S, 40°35′37″ W) and Praia Brava, Ubatuba, SP, Brazil (24°37′47″ S, 45°12′6″ W). Samples were kept at −20 °C prior to extraction. Voucher specimens were deposited at the Herbário Maria Eneida P. K. Fidalgo, São Paulo (SP 399.806, SP 400.905, SP 400.198, SP 400.202, SP 401.375 and SP 427.944).

### 3.3. Extract Preparation and Chromatographyc Analyses

Algal samples were lyophilized (Labconco, Freezone 13, Kansas City, MO, USA) and ground. Extracts were obtained by maceration of dry seaweed (30 g) with dichloromethane (DCM) 1:10 (*m/v*). The resultant extract was centrifuged at 10,000 rpm for 10 min, filtered, and concentrated under reduced pressure. This procedure was repeated three times.

The final extract (2.13 g; 7.10%) was analyzed employing thin-layer chromatography (TLC) and gas chromatography-mass spectrometry (GC-MS).

Plates of silica gel 60 (Merck, Darmstadt, Germany) with a UV indicator (254 nm) (20 cm × 10 cm × 0.1 mm) were utilized for TLC analyses. Samples were eluted with dichloromethane/methanol 0.5% and derivatized with Komarowsky reagent (solution of 50% ethanolic sulfuric acid and 2% methanolic *p*-hydroxybenzaldehyde, 1:10 (*v/v*), mixed shortly before use, heated at 100 °C for 3–5 min) [24].

A chromatographic column HP-5MS phase (5%-phenyl)-dimethylpolysiloxane (30 m × 0.25 µm × 0.25 µm) was used for GC-MS analyses and runs were programmed as follows: injector temperature 220 °C; oven temperature from 60 °C to 240 °C with 3 °C/min ramp and kept at 240 °C for 40 min; interface and detector temperature 240 °C; injection volume of 1 µL with split 5; helium was the carrier gas with a flowrate of 1 mL min^−1^; ionization method was electron impact. We used a detector mass range of 40–1000 *m/z* with access to NIST 08 and NIST 08-s libraries, available in GCMS-QP2010 Plus (Shimadzu, Kyoto, Japan) and NIST Mass Spectral Search Program from NIST/EPA/NIH Mass Spectral Library Version 2.0.

### 3.4. Biomonitored Fractioning

Fractionation of *L. dendroidea* extract was performed using liquid column chromatography (LCC) at ambient pressure with the column (1.50 m × 3 cm) filled with silica gel 60 (0.063–0.2 mm/70–230 mesh ASTM) employing isocratic elution with DCM:CH_3_OH (99.5:0.5). Resultant fractions were analyzed by TLC and GC-MS (see Section 3.3), and their molluscicidal (see Section 3.6) and cercaricidal (see Section 3.7) activities evaluated. Active fractions were de-replicated and purified employing HPLC coupled to an Ultra II column (250 × 10 mm i.d.) packed with silica (5 µm particle diameter; 100 Å average pore size, Restek, Bellefonte, PA, USA) with direct injection. The mobile phase consisted of petroleum ether/acetone (elution program: 0−30 min: 95−70% petroleum ether, held at 70% over 10 min, 70–95% over 15 min, held at 95% over 5 min; flow rate 3 mL/min) to yield 39 fractions. Fraction 12, (−)-elatol (**1**), was identified by comparison of its spectroscopic data with previously reported spectral data [14,25,26].

(−)-Elatol (**1**). Isolated as clear oil (4.48 mg, 0.22%) C_15_H_22_BrClO). ^1^H NMR (CDCl_3_, 400 MHz) δ = 1.07 (s, H-12), 1.08 (s, H-13), 1.64 (m, H-5), 1.70 (br s, H-15), 1.81 (m, H-5), 1.84 (m, H-4), 1.96 (m, H-4), 2.36 (br d 17.7 Hz, H-1), 2.56 (m, H-1), 2.50 (dd 2.7, 14.5 Hz, H-8), 2.63 (dm, 14.5 Hz, H-8), 4.15 (q, 3.0, 6.3 Hz, H-9), 4.61 (d, 2.9 Hz, H-10), 4.80 (br s, H-14), 5.13 (br s, H-14). ^13^C NMR (CDCl_3_, 125 MHz) δ = 19.4 (CH_3_-C15), 20.7 (CH_3_-C12), 24.2 (CH_3_-C13), 25.6 (CH_2_-C5), 29.3 (CH_2_-C4), 38.0 (CH_2_-C8), 38.1 (CH_2_-C1), 43.1 (C-C11), 49.1 (C-C6), 70.9 (CH-C10), 72.1 (CH-C9), 115.9 (CH_2_-C14), 124.1 (C-C3), 128.1 (C-C2), 140.7 (C-C7). EI-MS *m/z* (rel int %) 334 (0.1), 319 (2), 317 (1), 299 (3), 297 (3), 281 (2), 253 (8), 237 (32), 235 (76), 207 (24), 200 (10), 199 (41), 91 (59), 85 (100), 69 (50), 41 (51). ${\left[\alpha \right]}_{D}^{20}$ = −89.5° (*c* 0.1, MeOH). FTIR *ν*_max_ 3400–3600, 3100, 2946, 1641, 1429, 1338, 1085, 896, 810, 762, 620 cm^−1^. The 1D and 2D NMR spectra, and MS and IR spectra are available as Figures S1–S6 (Supplementary Materials).

### 3.5. Sesquiternene Purification and Identification

Isolation of sesquiterpenes **2** and **3** was achieved by employing a LC-8A Preparative Liquid Chromatography (Shimadzu, Kyoto, Japan) consisting of a binary pump module, a FRC-10A fraction collector, and a SPD-20A prominence UV–Vis detector coupled to a computer running Shimadzu software.

Separations of extracts were made using an Ultra column (250 × 21.2 mm i.d.) packed with silica (5 µm particle diameter; 100 Å average pore size, Restek, Bellefonte, PA, USA) with a silica gel ultrapure (60–200 µm, 60 Å average pore size) (Acrōs Organics, (Geel, Antwerp, Belgium) guard column. The mobile phase consisted of petroleum ether/acetone; gradient elution 95–70% over 80 min, held at 70% over 10 min, and 70–95% over 20 min. The flow rate was 3 mL/min, with detection by UV at 210 and 254 nm. Fractions were collected automatically every 2 min.

Further separations of selected fractions were made employing an Ultra II column (250 × 10 mm i.d.) packed with silica (5 µm particle diameter; 100 Å average pore size, Restek, Bellefonte, PA, USA) with direct injection. The mobile phase consisted of petroleum ether/acetone; gradient elution 95–70% over 40 min, held at 70% over 10 min, and 70–95% over 10 min. The flow rate was 2.5 mL/min, with detection by UV at 210 and 254 nm. Fractions were collected manually based on direct observation of chromatographic peaks. Monitoring by ^1^H NMR afforded the main sesquiterpenes (−)-rogiolol (**2**) and (+)-obtusol (**3**), which were identified by comparison of their spectroscopic data with previously reported spectral data [18,27,28,29,30,31,32,33].

(−)-Rogiolol (**2**). Isolated as clear oil (26.35 mg, 1.32%, C_15_H_23_Br_2_ClO). ^1^H NMR (CDCl_3_, 400 MHz, room temperature) δ = 5.38 (s, H-12), 5.04 (s, H-12), 4.71 (br d, 8.0 Hz, H-2), 4.46 (d, 3.1 Hz, H-8), 4.11 (br s, H-9), 2.48 (dd, 2.8, 14.2 Hz, H-10), 2.61 (br d, 14.2 Hz, H-10), 2.19 (dd, 3.4, 3.6 Hz, H-6), 2.16 (m, H-6), 2.09 (dd, 12.9, 14.3 Hz, H-3), 1.93 (m, H-5), 1.75 (dd, 3.2, 14.2 Hz, H-5), 1.69 (s, H-15), 1.08 (s, H-13), 1.07 (s, H-14). ^13^C NMR (CDCl_3_, 125 MHz) δ = 20.6 (CH_3_-C14), 23.9 (CH_3_-C15), 24.1 (CH_3_-C13), 25.5 (CH_2_-C5), 38.5 (CH_2_-C3), 38.6 (CH_2_-C10), 38.7 (CH_2_-C6), 44.1 (C-C7), 50.9 (C-C4), 61.0 (CH-C2), 70.1 (CH-C8), 71.7 (C-C1), 71.9 (CH-C9), 117.7 (CH_2_-C12), 141.2 (C-C11). APCI-MS *m/z* [M]^−^ 414.9648, *m/z* [M − H]^−^ 412.9665 (calc. for [M − H]^−^ 412.9710. ${\left[\alpha \right]}_{D}^{20}$ = −3.67° (*c* 0.6, MeOH). FTIR *ν*_max_ 3500, 3100, 1650, 1434, 1380, 1198, 1093, 905, 815, 736, 618 cm^−1^. The 1D and 2D NMR spectra, and MS and IR spectra are available as Figures S7–S14 (Supplementary Materials).

(+)-Obtusol (**3**). Isolated as a yellowish oil (5.5 mg, 0.26%, C_15_H_23_Br_2_ClO). ^1^H NMR (CDCl_3_, 500 MHz) δ = 1.08 (s, H-13), 1.08 (s, H-14), 1.74 (br dd, 3.2, 12.5 Hz, H-5), 1.83 (s, H-15), 1.94 (t, 13.4, H-6), 2.29 (dt, 3.5, 13.4 Hz, H-3), 2.35 (t, 7.48 Hz, H-6), 2.50 (br dd, 2.3, 3.2 Hz, H-10), 2.62 (d, 14.1, H-10), 4.12 (br s, H-9), 4.47 (br d, 2.8, H-8), 4.72 (br s, H-2), 5.06 (s, H-12), 5.39 (s, H-12). ^13^C NMR (CDCl_3_, 125 MHz) δ = 20.5 (CH_3_-C14), 23.9 (CH_3_-C15). 24.1 (CH_3_-C13), 25.6 (CH_2_-C5), 37.1 (CH_2_-C6), 38.5 (C-C11), 40.5 (CH_2_-C3), 44.3 (C-C7), 50.3 (C-C4), 67.6 (CH-C2), 68.1(C-C1), 70.1 (CH-C8), 71.9 (CH-C9), 117.9 (CH_2_-C12), 141.2 (C-C11). EI-MS *m/z* (rel int %): 320 (3), 319 (20), 318 (13), 317 (78), 315 (61), 299 (19), 297 (19), 281 (11), 279 (11), 253 (4), 237 (7), 235 (14), 217 (8), 199 (47), 157 (38), 143 (33), 133 (36), 119 (50), 107 (74), 105 (57), 93 (47), 91 (45), 85 (100), 55 (44), 41 (35). ${\left[\alpha \right]}_{D}^{20}$ = +10.21° (*c* 0.65, CHCl_3_). FTIR *ν*_max_ 3587, 2968, 2924, 1638, 1433, 1088, 808, 732 cm^−1^. The 1D and 2D NMR spectra, and MS and IR spectra are available as Figures S15–S19 (Supplementary Materials).

### 3.6. Molluscicidal Activity

Groups of egg masses, with at least 50 embryos each, were placed in 12-well culture plates and kept at 25 ± 2 °C. The organisms were exposed to solutions of extracts (100 mg L^−1^)/fractions (50 mg L^−1^)/isolated compounds (20 mg L^−1^) for 24 h. After this period, they were transferred to another plate containing dechlorinated water for seven days. Dechlorinated water/DMSO (3%) was used as the negative control. Organisms were evaluated daily, employing a stereoscope microscope, for lethality or teratogenic effects. All tests were conducted in triplicate.

### 3.7. Cercaricidal Activity

Samples of 50 cercariae mL^−1^ of dechlorinated water (2 mL/group) were placed in 24-well culture plates and kept at room temperature of 25 ± 2 °C. Cercariae were exposed for 120 min to a solution of extracts (100 mg L^−1^), fractions (50 mg L^−1^), and isolated compounds (20 mg L^−1^). Dechlorinated water/DMSO (3%) was used as the negative control. Larvae were evaluated at 5, 15, 30, 60, and 120 min after exposure employing a stereoscopic microscope; those motionless at the bottom of the plate were considered dead.

### 3.8. SchistosomicidalActivity in Adult Worms

Adult worms were recovered through portal perfusion from hamsters 42 days after infection. Five coupled male and female worms were exposed to the test compounds; praziquantel was used as the positive control and DMSO as the negative control. Worms were maintained in 24-well culture plates at 37 °C and 5% CO_2_, and monitored after 2 h and then every 24 h thereafter for 96 h for motility, morphological alterations, and reproduction [10]. Experimental procedures were employed according to accepted principles of animal welfare in experimental science (CEUA N 5042140818).

### 3.9. Statistical Analysis

For survival analysis, it is important to note that each sample unit registers only one result. That is, the samples have no intersection among doses. For each dose, we estimated the population proportion for that dose by the sample proportion of that dose. The survival units could be considered as having been generated by beta probability densities (a distribution defined on the unit interval [0;1]). For simplicity, we transformed the proportions to their log-odds. Log-odds of beta densities have approximately normal distributions with mean and variance obtained by the mathematical functions digamma and trigamma, respectively.

The formal explanations of the method above can be found in Aitchison [33]. The statistical work including the graphs was performed with the Excel, from Microsoft Office 365.

## Figures and Tables

**Table 1:** Activity of seaweed derived halogenated sesquiterpenes on *S. mansoni* and *B. glabrata*.

Models Tested	(−)-Elatol	Rogiolol	Obtusol
*S. mansoni* worms			
Viability	−	+++	+
Reproduction	+++	+++	+++
*S. mansoni* cercariae	+++	+++	+++
*B. glabrata* embryos	+++	+++	+++

Concentrations for *S. mansoni* adult worms = 50 µg/mL; *S. mansoni* cercariae and *B. glabrata* embryos = 25 µg/mL. Activity: (+++) = 100% mortality; (+) = <50% mortality; (−) = non active.

**Table 2:** Activity of (−)-elatol against cercariae of *S. mansoni*.

Concentration (µg mL^−1^)	Inhibition * of Cercariae after Fixed Times in min				
	5	15	30	60	120
Dechlorinated water	−	−	−	−	−
Dechlorinated water with DMSO 1%	−	−	−	−	−
3.13	+	+	+	+	+
6.25	++	+	+	+	+
12.5	+++	+++	+++	+++	+++
25	+++	+++	+++	+++	+++
50	+++	+++	+++	+++	+++
100	+++	+++	+++	+++	+++

* (+++) 100% of cercariae motionless at the bottom of the test plate, (++) between 50% and 100% of cercariae motionless at the bottom of the test plate, (+) between 10% and 50% of cercariae motionless at the bottom of the test plate, and (−) lack of larvicidal activity with ≥90% of larvae swimming.

## Supplementary Materials

The following are available online at https://www.mdpi.com/article/10.3390/md20020111/s1, Figure S1. ^1^H NMR spectrum (400 MHz, CDCl_3_) of (−)-elatol, Figure S2. ^13^C NMR spectrum (100 MHz, CDCl_3_) of (−)-elatol, Figure S3. HSQC NMR spectra (400 MHz, CDCl_3_) of (−)-elatol, Figure S4. HMBC NMR spectrum (400 MHz, CDCl_3_) of (−)-elatol, Figure S5. Mass spectra obtained by EI-MS (70 eV) of (−)-elatol, Figure S6. Infrared spectra of (−)-elatol, Figure S7. ^1^H NMR spectrum (400 MHz, CDCl_3_) of rogiolol, Figure S8. ^13^C NMR spectrum (100 MHz, CDCl_3_) of rogiolol, Figure S9. HSQC NMR spectrum (400 MHz, CDCl_3_) of rogiolol, Figure S10. HMBC NMR spectrum (400 MHz, CDCl_3_) of rogiolol, Figure S11. COSY NMR spectrum (400 MHz, CDCl_3_) of rogiolol, Figure S12. APCI-MS fragmentation of rogiolol, Figure S13. Mass spectrum obtained by EI-MS (70 eV) of rogiolol, Figure S14. Infrared spectrum of rogiolol, Figure S15. ^1^H NMR spectrum (500 MHz, CDCl_3_) of obtusol, Figure S16. ^13^C NMR spectrum (125 MHz, CDCl_3_) of obtusol, Figure S17. HMBC NMR spectrum (500 MHz, CDCl_3_) of obtusol, Figure S18. EI-MS fragmentation of obtusol, Figure S19. Infrared spectrum of obtusol, Figure S20. Full chromatogram of *Laurencia dendroidea* extract in dichloromethane obtained by GC-MS.
