# Supplementary material for: Algal-Derived Halogenated Sesquiterpenes from Laurencia dendroidea as Lead Compounds in Schistosomiasis Environmental Control
Source: Mar Drugs. 2022 Jan 29;20(2):111. doi: 10.3390/md20020111 (PMC8874501; doi:10.3390/md20020111)
Supplement: Supplementary file 1 [file marinedrugs-20-00111-s001.zip › marinedrugs-1512211-supplementary.pdf]

SUPPLEMENTARY MATERIAL

Algal-Derived Halogenated Sesquiterpenes from *Laurencia dendroidea* as Lead Compounds in Schistosomiasis Environmental Control

Guilherme Senna dos Santos [1], Patrícia Aoki Miyasato [1], Erika Mattos Stein [2], Pio Colepicolo [2], Anthony D. Wright [3,4], Carlos Alberto de Bragança Pereira [5], Miriam Falkenberg [6] and Eliana Nakano [1,*]

[1] Laboratory of Parasitology, Butantan Institute, São Paulo, SP CEP 05503-000, Brazil; gsennasantos@gmail.com (G.S.d.S.); patricia.aoki@butantan.gov.br (P.A.M.)
[2] Biochemistry Department, Chemistry Institute, USP—Universidade de São Paulo, São Paulo, SP 05508-00, Brazil; stein.erika.m@gmail.com (E.M.S.); piocolep@iq.usp.br (P.C.)
[3] DKI College of Pharmacy, University of Hawaii at Hilo, Hilo, HI 96720, USA; adwhawaii@gmail.com
[4] Right Consulting, 15 Amauulu Road, Hilo, HI 96720, USA
[5] Institute of Mathematics and Statistics, USP—Universidade de São Paulo, São Paulo, SP CEP 05503-000 Brazil; cadebp@gmail.com
[6] Department of Pharmaceutical Sciences, Federal University of Santa Catarina, Florianópolis, SC 88040-970, Brazil; miriam.falkenberg@gmail.com
* Correspondence: eliana.nakano@butantan.gov.br; Tel.: +55-11-2627-9770

SUPPLEMENTARY MATERIAL 1
Figure S1. ^{1}H NMR spectrum (400 MHz, $CDCl_3$) of (-)-Elatol 3
Figure S2. ^{13}C NMR spectrum (100 MHz, $CDCl_3$) of (-)-Elatol 3
Figure S3. HSQC NMR spectra (400 MHz, $CDCl_3$) of (-)-Elatol 4
Figure S4. HMBC NMR spectrum (400 MHz, $CDCl_3$) of (-)-Elatol 5
Figure S5. Mass spectrum obtained by EI-MS (70 eV) of (-)-Elatol 5
Figure S6. Infrared Spectrum of (-)-Elatol 6
Figure S7. ^{1}H NMR spectrum (400 MHz, $CDCl_3$) of Rogiolol 7
Figure S8. ^{13}C NMR spectrum (100 MHz, $CDCl_3$) of Rogiolol 7
Figure S9. HSQC NMR spectra (400 MHz, $CDCl_3$) of Rogiolol 9
Figure S10. HMBC NMR spectrum (400 MHz, $CDCl_3$) of Rogiolol... 9
Figure S11. COSY NMR spectrum (400 MHz, $CDCl_3$) of Rogiolol 10
Figure S12. APCI-MS fragmentation of Rogiolol... 10
Figure S13. Mass spectrum obtained by EI-MS (70 eV) of Rogiolol 10
Figure S14. Infrared Spectrum of Rogiolol 11
Figure S15. ^{1}H NMR spectrum (500 MHz, $CDCl_3$) of Obtusol 11
Figure S16. ^{13}C NMR spectrum (125 MHz, $CDCl_3$) of Obtusol.. 12
Figure S17. HMBC NMR spectrum (500 MHz, $CDCl_3$) of Obtusol 12
Figure S18. EI-MS fragmentation of Obtusol 12
Figure S19. Infrared Spectrum of Obtusol 13
Figure S20. Full chromatogram of *Laurencia dendroidea* extract in dichloromethane obtained by GC-MS.....13

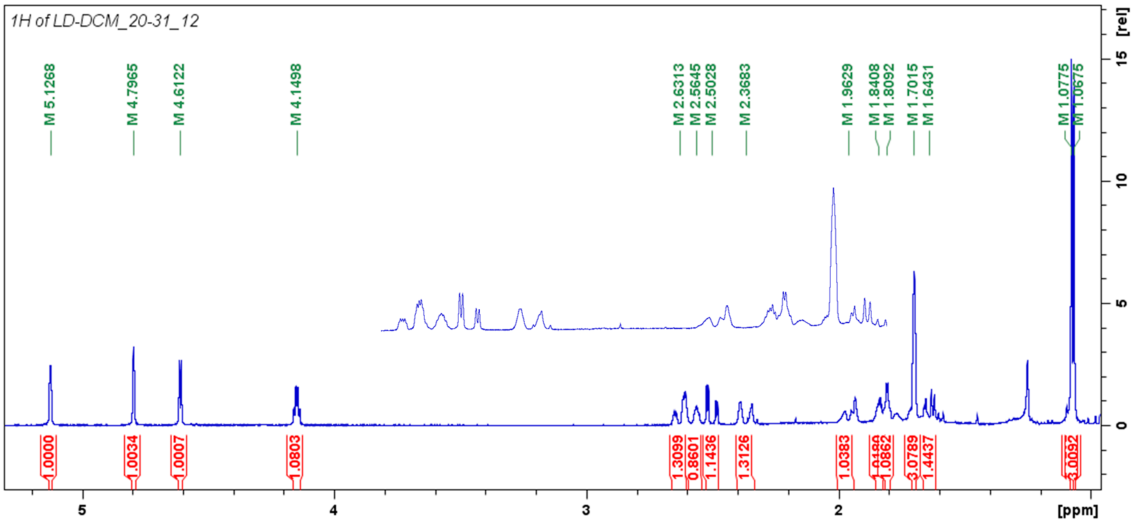

Figure S1. [1]**H NMR spectrum (400 MHz, $CDCl_3$) of (-)-Elatol**

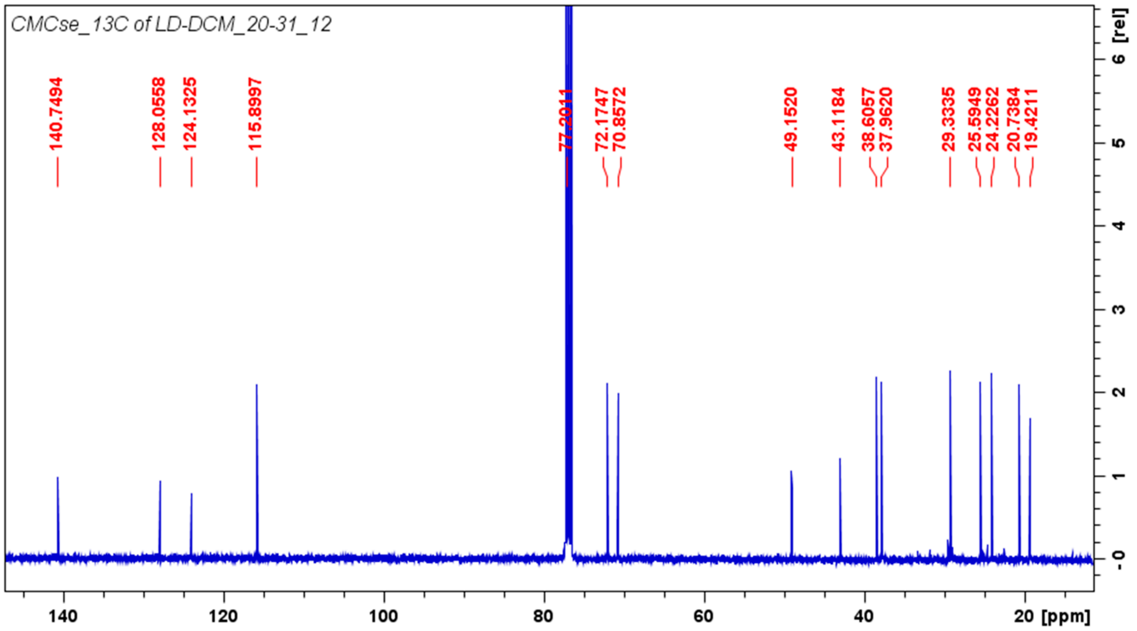

Figure S2. [13]**C NMR spectrum (100 MHz, $CDCl_3$) of (-)-Elatol**

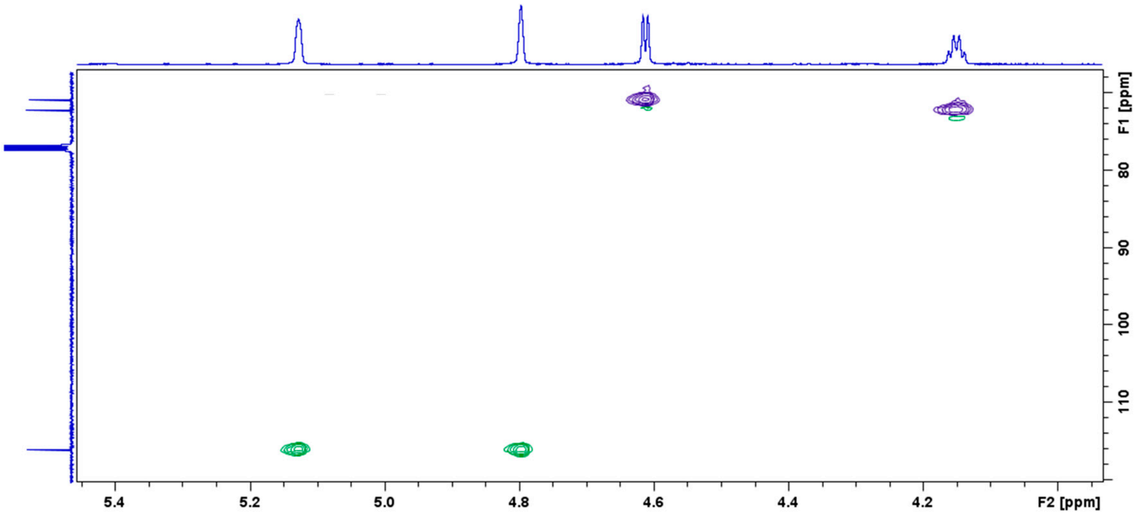

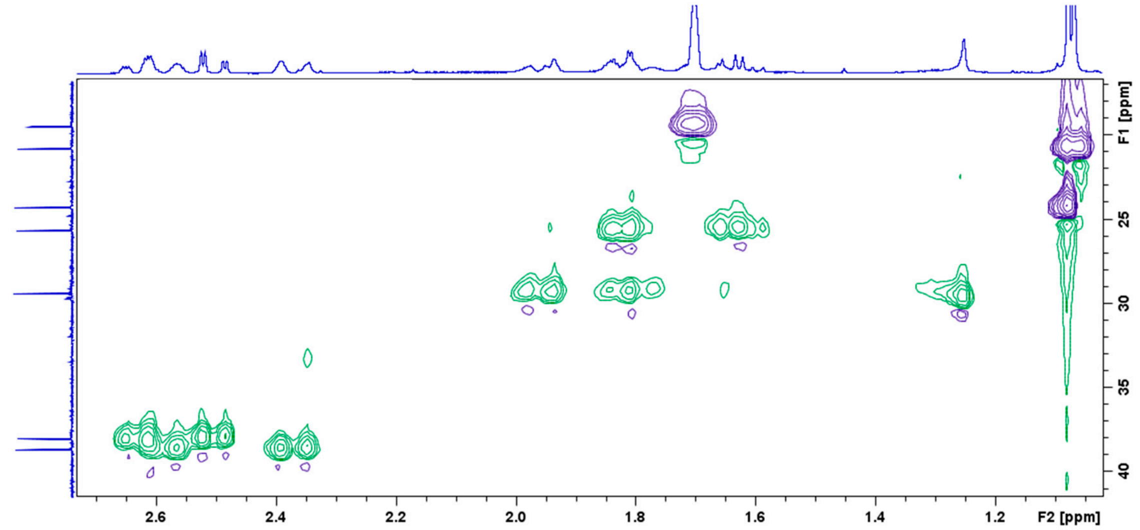

Figure S3 HSQC NMR spectra (400 MHz, $CDCl_3$) of (-)-Elatol

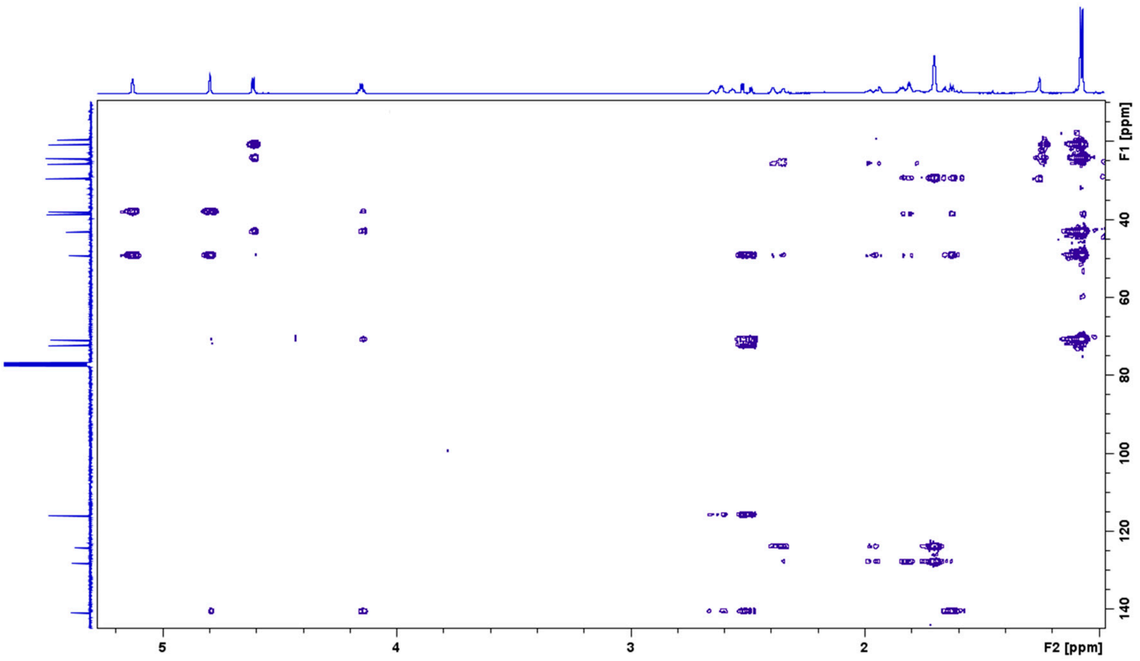

Figure S4. HMBC NMR spectrum (400 MHz, $CDCl_3$) of (-)-Elatol

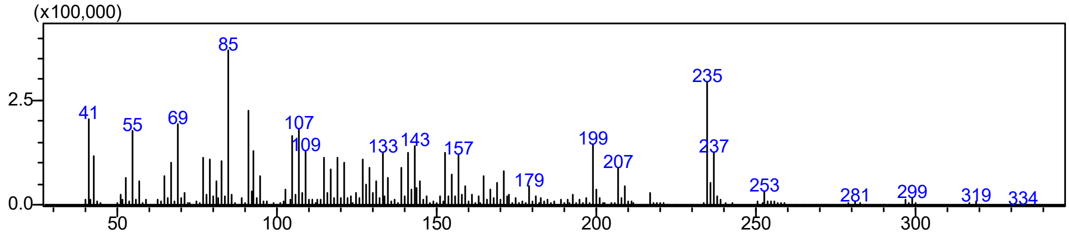

Figure S5. Mass spectrum obtained by EI-MS (70 eV) of (-)-Elatol

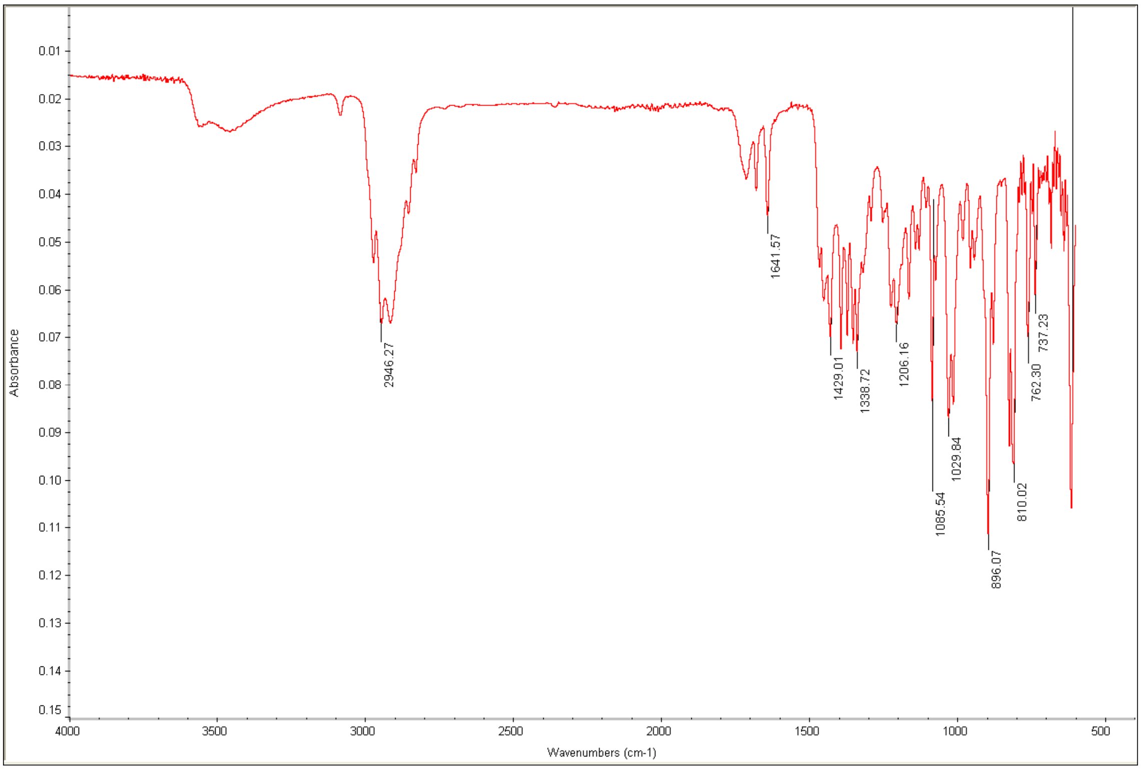

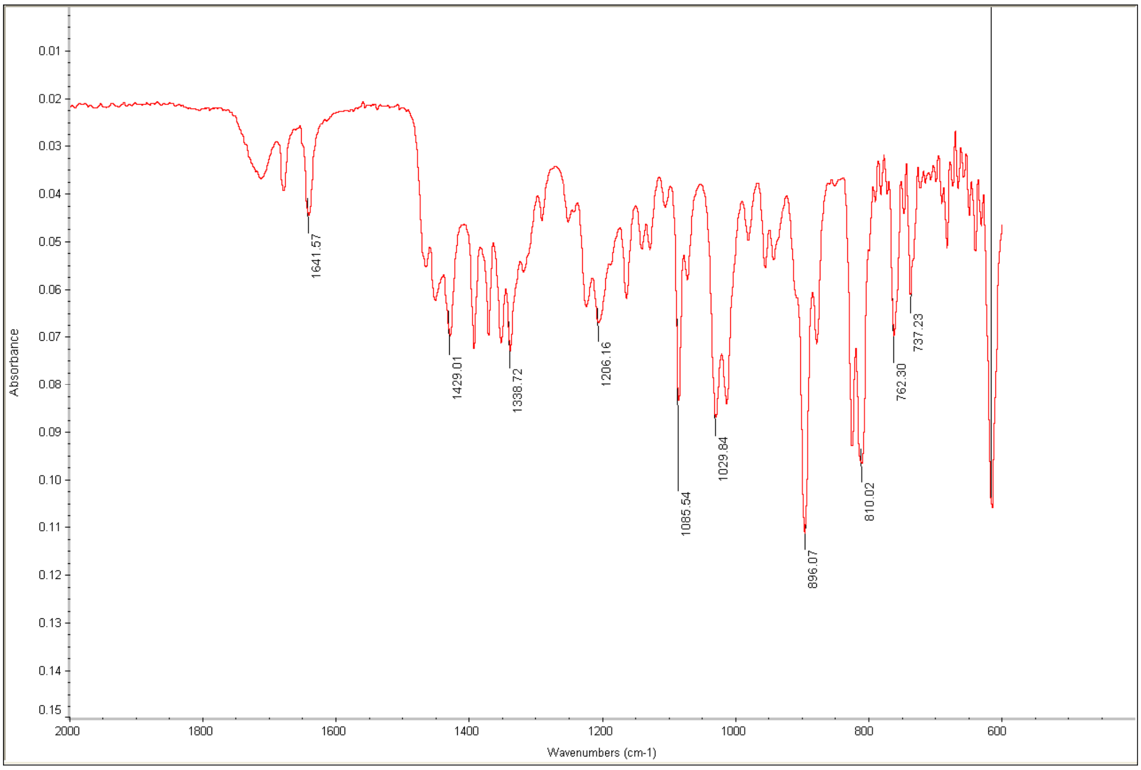

Figure S6. Infrared Spectrum of (-)-Elatol

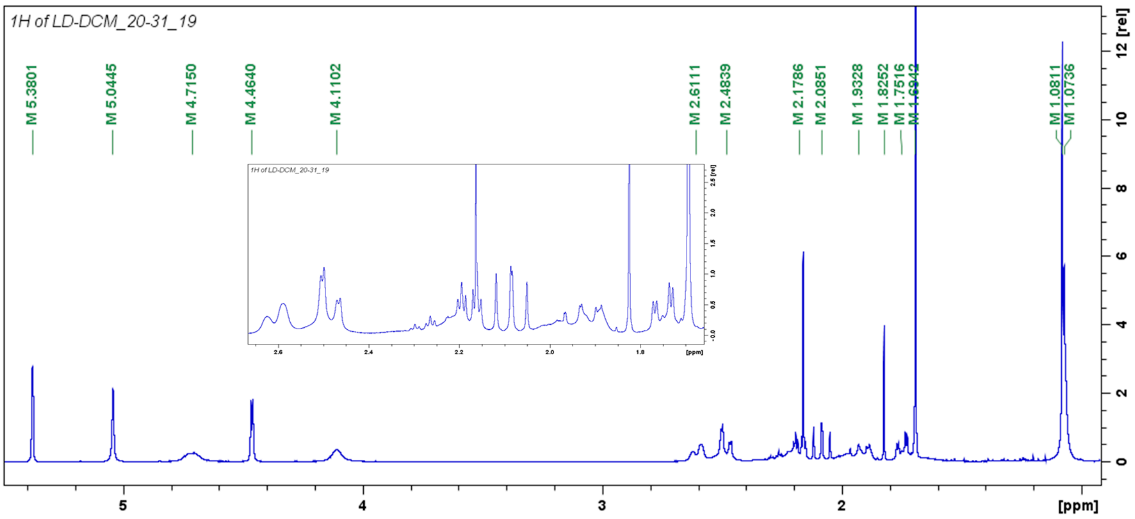

Figure S7. ^{1}H NMR spectrum (400 MHz, $CDCl_3$) of Rogiolol

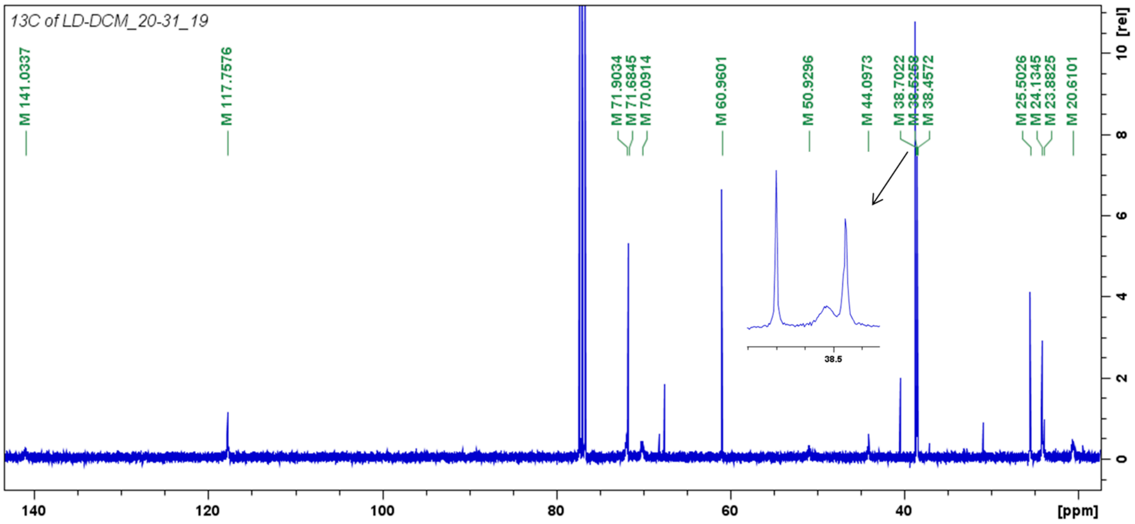

Figure S8. ^{13}C NMR spectrum (100 MHz, $CDCl_3$) of Rogiolol

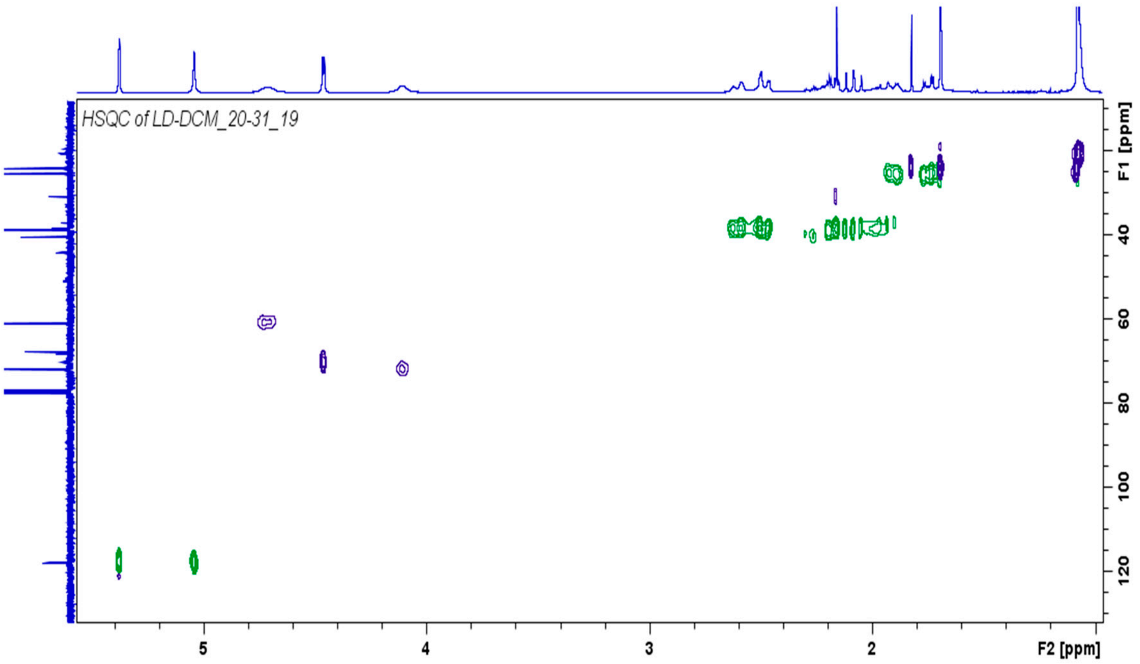
HSQC of LD-DCM_20-31_19
F1 [ppm]
F2 [ppm]

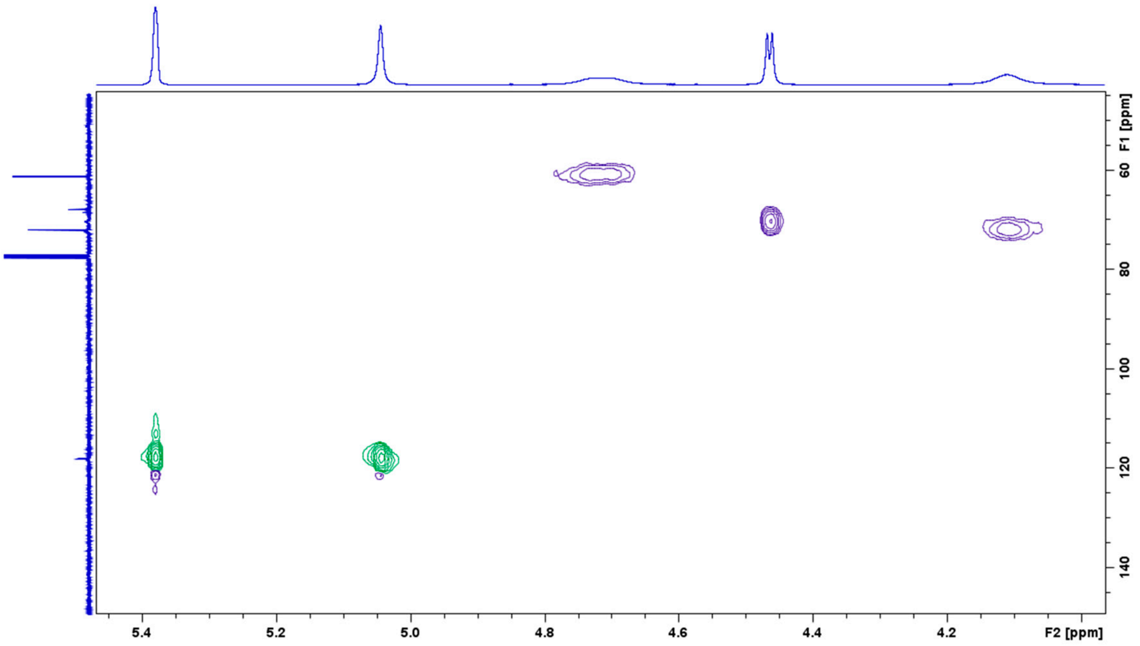
F1 [ppm]
F2 [ppm]

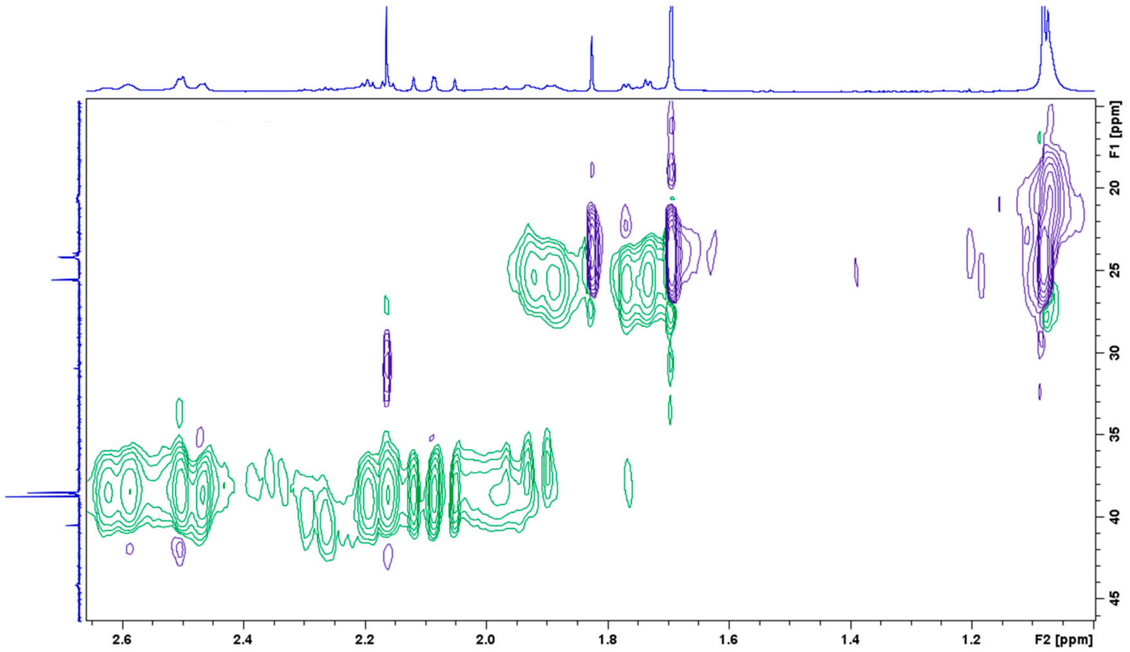

Figure S9. HSQC NMR spectra (400 MHz, $CDCl_3$) of Rogiolol

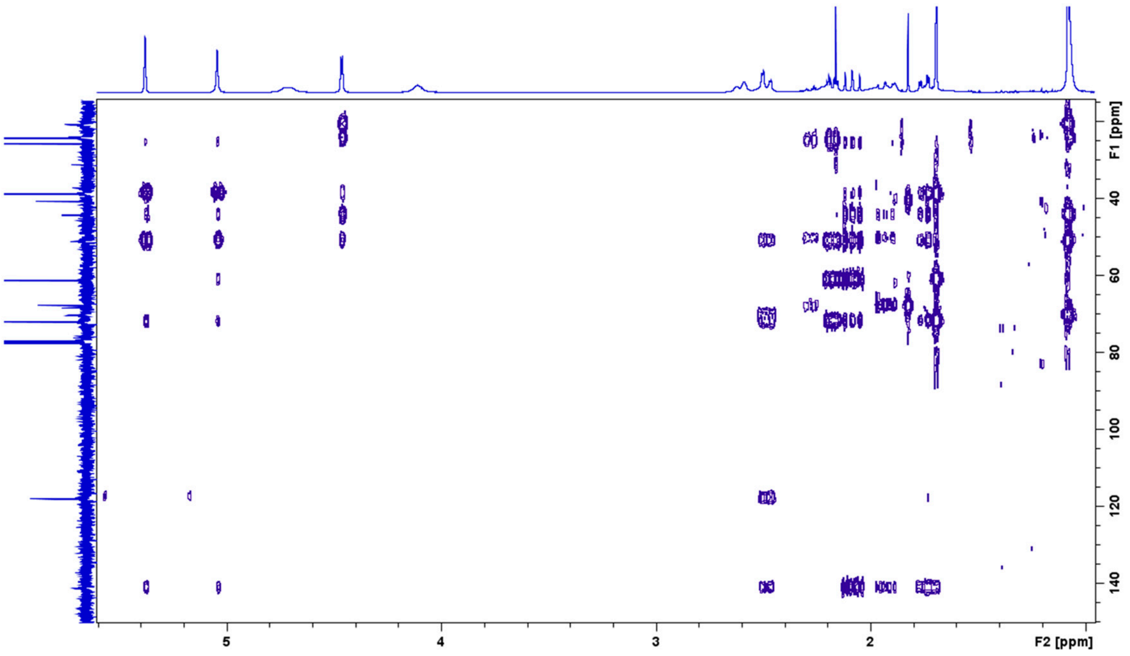

Figure S10. HMBC NMR spectrum (400 MHz, $CDCl_3$) of Rogiolol

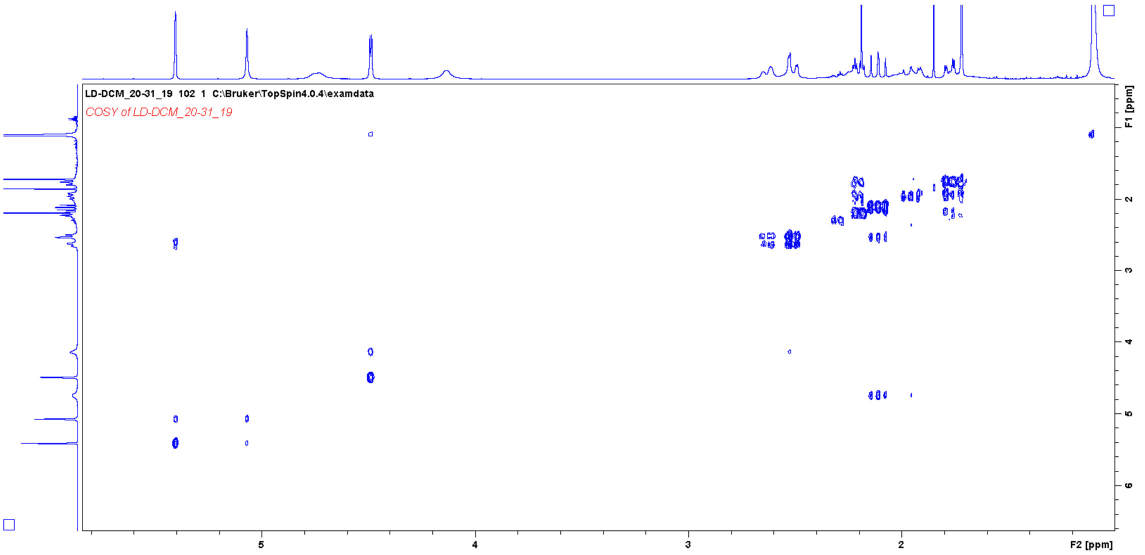

Figure S11. COSY NMR spectrum (400 MHz, CDCl₃) of Rogiolol

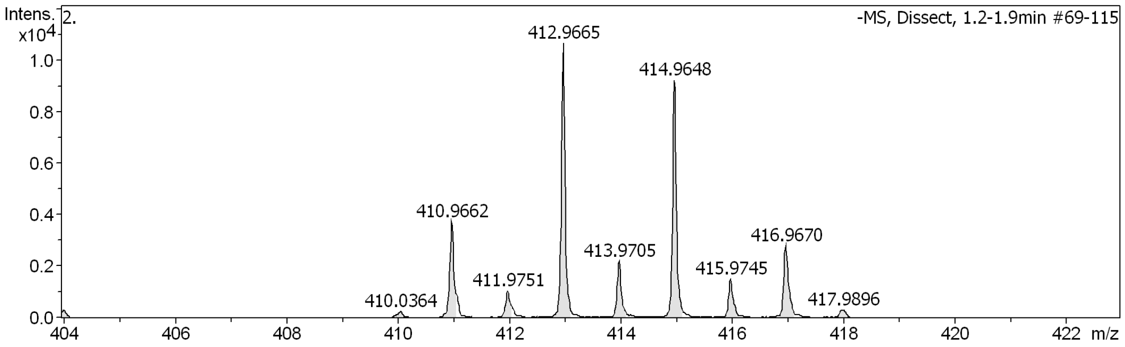

Figure S12. APCI-MS fragmentation of Rogiolol

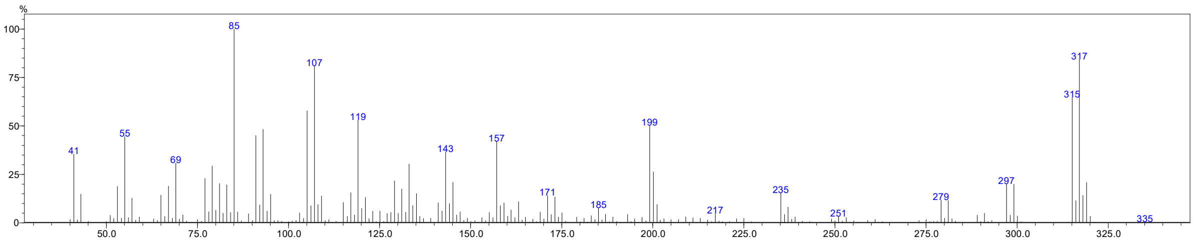

Figure S13. Mass spectrum obtained by EI-MS (70 eV) of Rogiolol

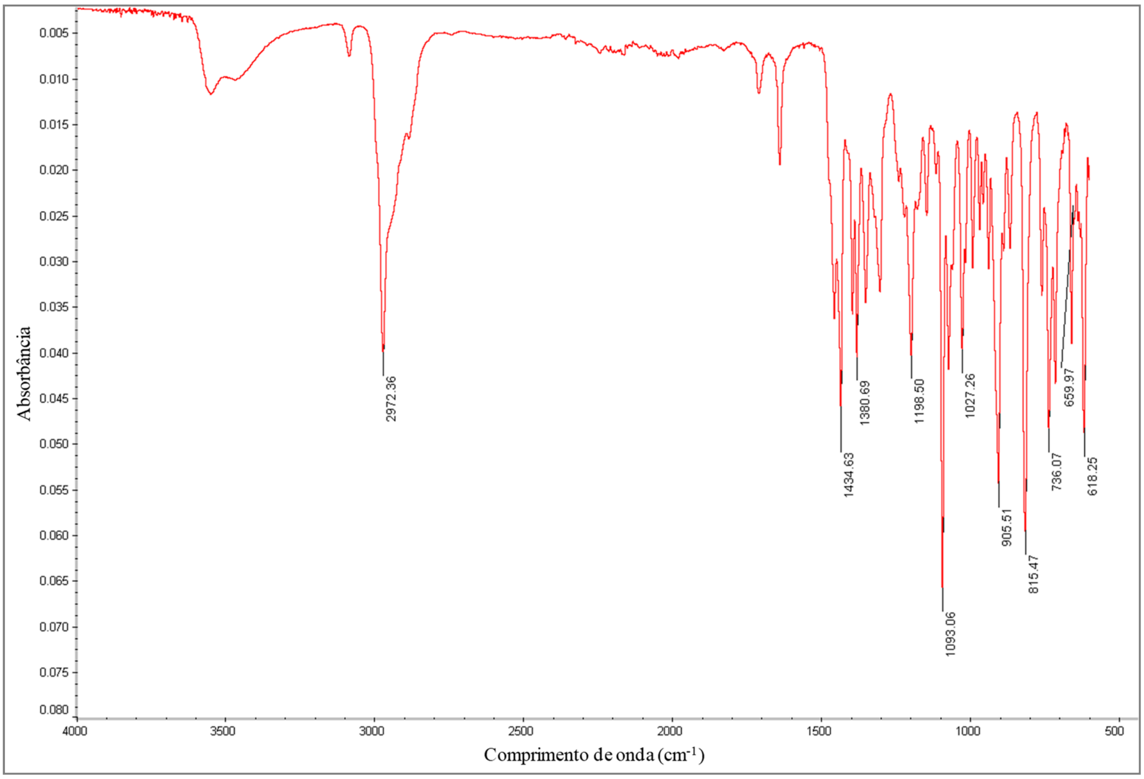

Figure S14. Infrared Spectrum of Rogiolol

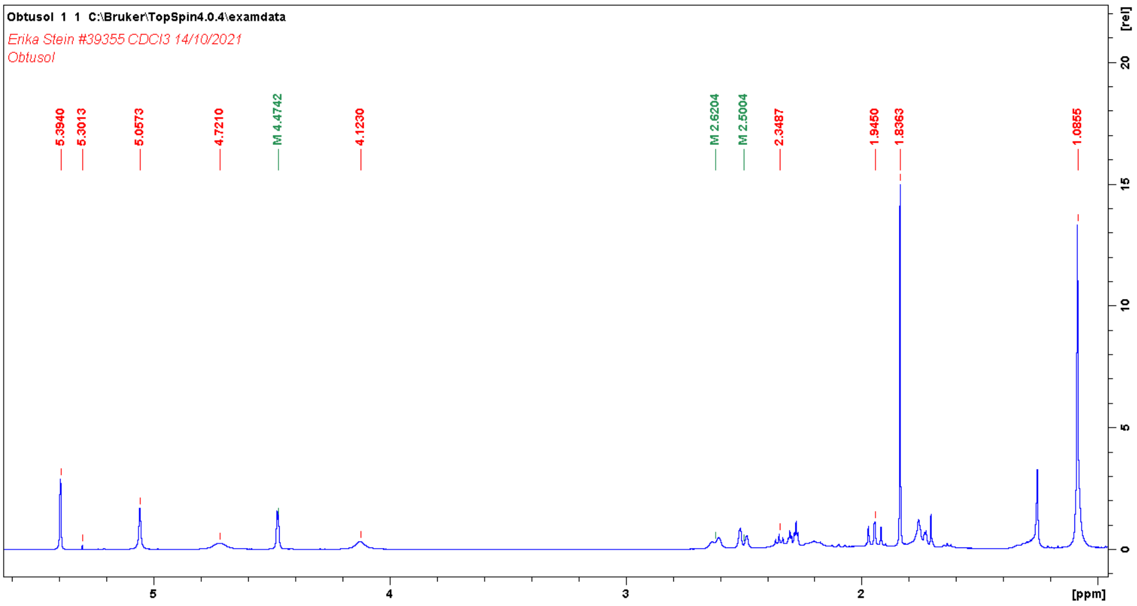

Figure S15. ^{1}H NMR spectrum (500 MHz, $CDCl_3$) of Obtusol

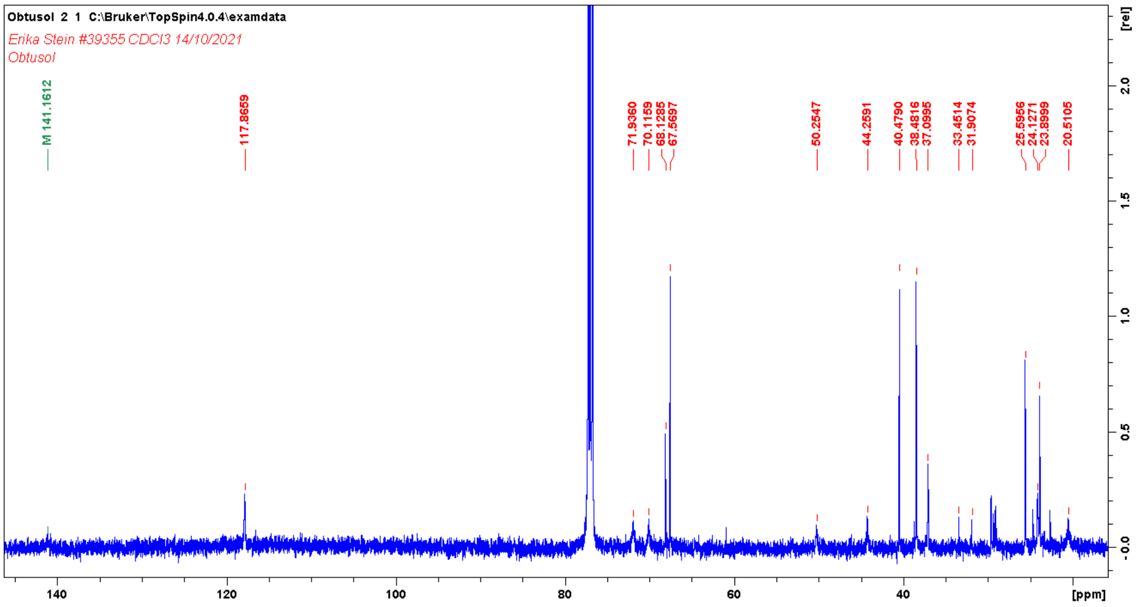

Figure S16. ^{13}C **NMR spectrum (125 MHz,** $CDCl_3$**) of Obtusol**

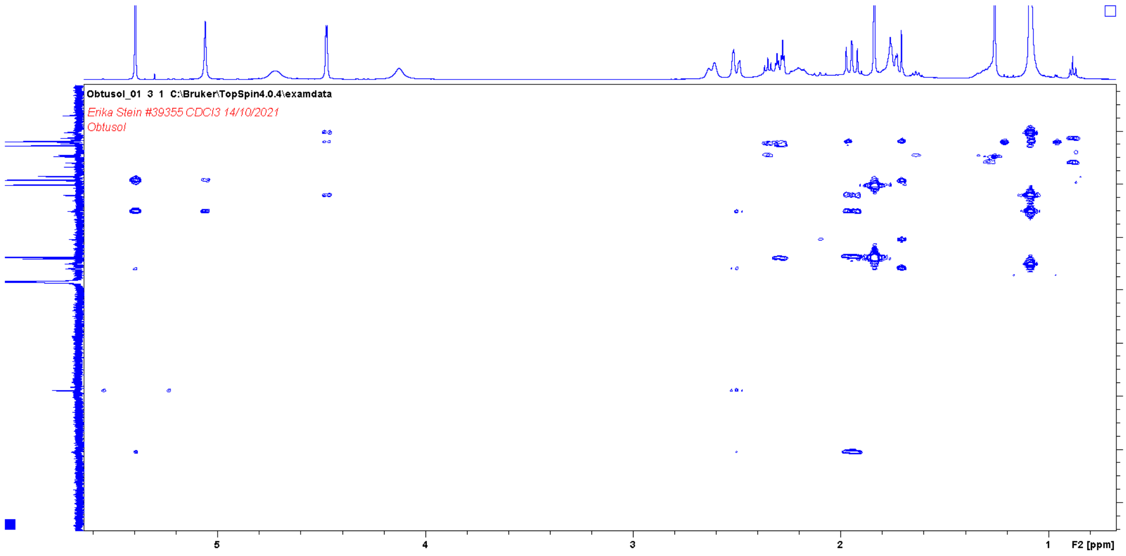

Figure S17. HMBC NMR spectrum (500 MHz, $CDCl_3$**) of Obtusol**

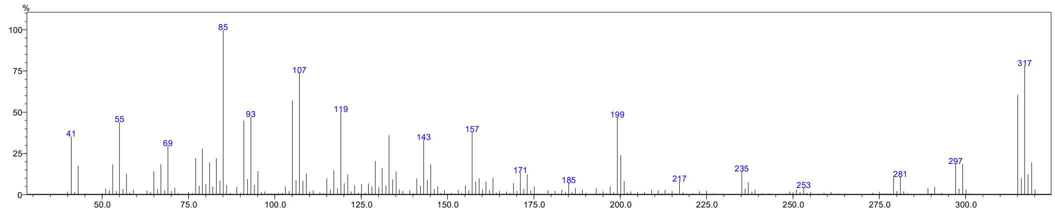

Figure S18. EI-MS fragmentation of Obtusol

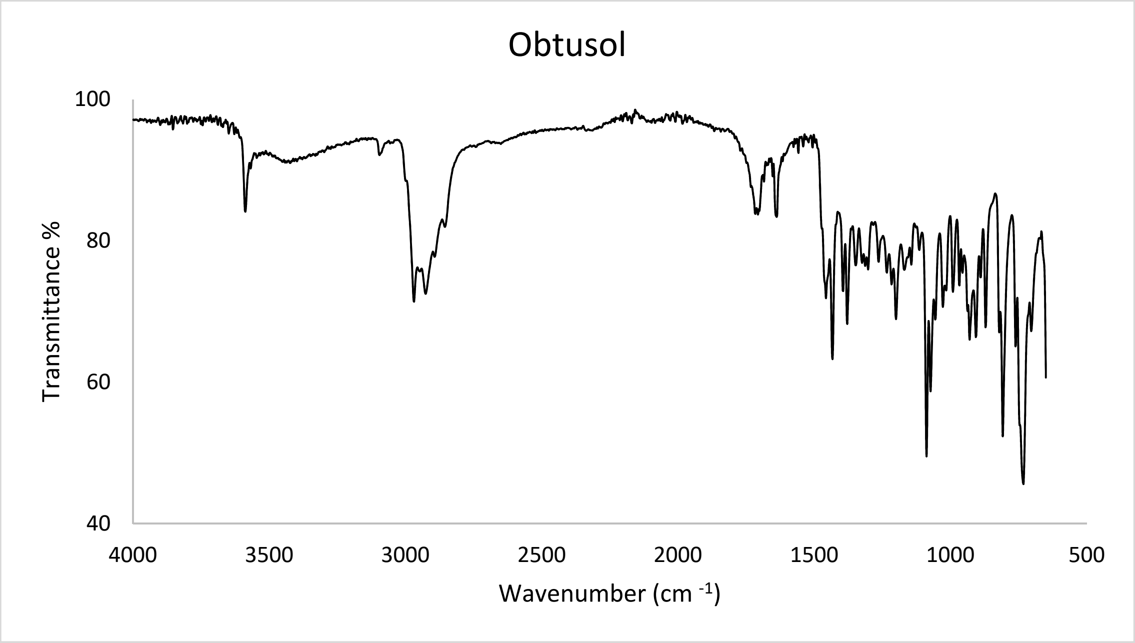

Figure S19. Infrared Spectrum of Obtusol

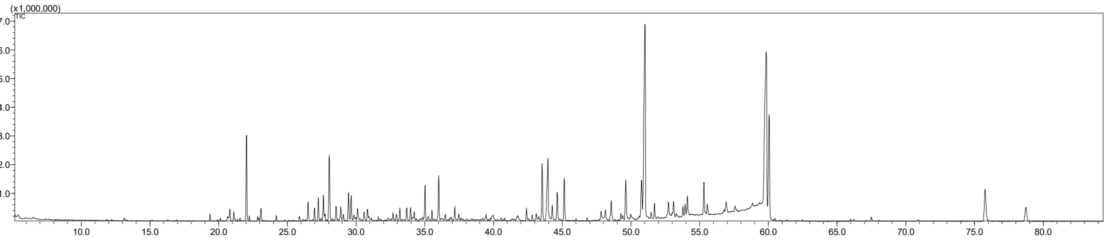

Figure S20. Full chromatogram of *Laurencia dendroidea* extract in dichloromethane obtained by GC-MS.
